# Communication methods and production techniques in fixed prosthesis fabrication: a UK based survey. Part 1: Communication methods

**DOI:** 10.1038/sj.bdj.2014.643

**Published:** 2014-09-26

**Authors:** J. Berry, M. Nesbit, S. Saberi, H. Petridis

**Affiliations:** 1 Clinical Lecturer, Department of Adult Oral Health, Institute of Dentistry, Barts and The London School of Medicine and Dentistry, Queen Mary University of London, London; 2 Senior Technical Instructor, Prosthodontic Unit, UCL Eastman Dental Institute, London; 3 Senior Lecturer, Department of Restorative Dentistry, Prosthodontics Unit, UCL Eastman Dental Institute, London

## Abstract

**Statement of the problem** The General Dental Council (GDC) states that members of the dental team have to 'communicate clearly and effectively with other team members and colleagues in the interest of patients'. A number of studies from different parts of the world have highlighted problems and confirmed the need for improved communication methods and production techniques between dentists and dental technicians.

**Aim** The aim of this study was to identify the communication methods and production techniques used by dentists and dental technicians for the fabrication of fixed prostheses within the UK from the dental technicians' perspective. The current publication reports on the communication methods.

**Materials and methods** Seven hundred and eighty-two online questionnaires were distributed to the Dental Laboratories Association membership and included a broad range of topics. Statistical analysis was undertaken to test the influence of various demographic variables.

**Results** The number of completed responses totalled 248 (32% response rate). The laboratory prescription and the telephone were the main communication tools used. Statistical analysis of the results showed that a greater number of communication methods were used by large laboratories. Frequently missing items from the laboratory prescription were the shade and the date required. The majority of respondents (73%) stated that a single shade was selected in over half of cases. Sixty-eight percent replied that the dentist allowed sufficient laboratory time. Twenty-six percent of laboratories felt either rarely involved or not involved at all as part of the dental team.

**Conclusion** This study suggests that there are continuing communication and teamwork issues between dentists and dental laboratories.

## Introduction

Prosthodontics is a discipline that requires a synergy between the dentist and dental technician in order to fabricate intraoral prostheses with acceptable fit, function and aesthetics.^1^,^2^,^3^ Proper communication between the two parties is very important because, in the majority of cases, the dental technicians are remotely located and usually never actually see the patient. The General Dental Council's (GDC) policy document *Principles of dental team working*^4^ states that: 'Members of the dental team will work effectively together', and also that members have to 'communicate clearly and effectively with other team members and colleagues in the interest of patients', and that 'if you ask a colleague to provide treatment, a dental appliance or clinical advice for a patient, make sure that your request is clear and that you give your colleague all the information they need'.^4^ The prerequisite for a proper prescription written by a qualified dentist has also been set in the Medical Devices Directive (MDD).^5^

A number of studies^6^,^7^,^8^,^9^,^10^,^11^,^12^ from different parts of the world have highlighted problems and confirmed the need for improved communication methods and production techniques between dentists and dental technicians, during the fabrication of fixed prosthodontic appliances. Problems seem to occur even within the same hospital setting.^13^,^14^ Communication issues have included lack of information regarding the prosthesis design and materials, the lack of understanding of the necessary technical steps and time required, and lack of proper shade communication.^6^,^7^,^8^,^9^,^10^,^11^,^12^ Most of the times, the final decision was left with the technician, without proper feedback. All of the above issues, compounded by the time pressure for completion of the restorations as noted in some studies,^8^,^11^ may explain the finding that many dental technicians feel insufficiently valued in the dental team.^11^,^12^,^15^

A number of studies^1^,^12^,^14^,^16^ have highlighted the lack of suitable instruction to dental undergraduates regarding effective communication between dentists and technicians, and the lack of knowledge regarding dental prosthesis fabrication at the time of qualification as the main factors for the re-occurring problems. This has led to the introduction of inter-professional education schemes in Australia.^17^

The last survey of UK-based dental laboratories was published in 2009,^12^ and suggested that the GDC had failed in its aims published in *The first five years; a framework for undergraduate dental education*,^18^ as serious communications issues were identified.^12^

The purpose of this cross-sectional study was to identify the communication methods and production techniques used by dentists and dental technicians for the fabrication of fixed prostheses within the UK from the dental technicians' perspective. The current publication reports on the communication methods.

## Materials and methods

A questionnaire was constructed to investigate communication methods and production techniques used between dentists and dental laboratories from the laboratories perspective. An effort was made to include a broad range of topics. At the same time elements of previously published research were incorporated in order to obtain meaningful results that would be comparable to past surveys. The final questionnaire consisted of 30 questions within the following subcategories: general information, communication methods, impression disinfection and suitability, production techniques, shade matching, and time and team management issues. The questionnaire was piloted among dental technicians both at UCL Eastman Dental Institute and in selected commercial laboratories.

The Dental Laboratories Association (DLA, Nottingham, UK) was approached and approved the use of their database of e-mail contacts (782 addresses).

A web-based survey tool, Opinio (ObjectPlanet Inc. Oslo, Norway), was utilised for the administration of the survey and assimilation of data. Settings were managed in order to ensure anonymity of respondents. The questionnaire weblink along with an introduction letter, were distributed through the DLA. The survey was 'live' for 6 weeks, and during that time the response rate was actively monitored and three e-mail reminders were sent.

The collected data was presented as descriptive statistics and analysed using Fisher's exact test, the Mann-Whitney test or the Spearman's rank correlation (SPSS 12.0; SPSS Inc, Chicago). P-values of less than 0.025 were regarded as statistically significant. A significance level of 2.5% was chosen rather than the conventional 5% to avoid spuriously significant results arising from multiple testing.

The null hypothesis was that factors such as the source of information used to answer the questionnaire, the location, and size of the dental laboratory, did not influence the communication methods and production techniques.

## Results

The number of responses totalled 248, which yielded a 32% response rate. Sixty-eight respondents answered only some of the questions. The results presented in this paper pertain to the subchapters of general information, communication methods, shade matching, and time and team management issues. The subchapters and questions along with the results in parentheses are depicted in Table 1.

The majority of the information (81%) used to answer the survey questions were sourced from memory and 19% of respondents used their laboratory records. Ninety percent of the respondents were based in England. This unequal distribution among England, Scotland, Wales and Northern Ireland did not permit any further analysis of this particular factor. The majority of dental laboratories (73%) completed work for less than 50 dentists and 13% worked with over 100 dentists. For analysis purposes the labs were grouped into three categories regarding size: small (43% working with up to 25 dentists), medium (38% working with 26-75 dentists) and large (19% working with 76+ dentists).

**Figure 1:**
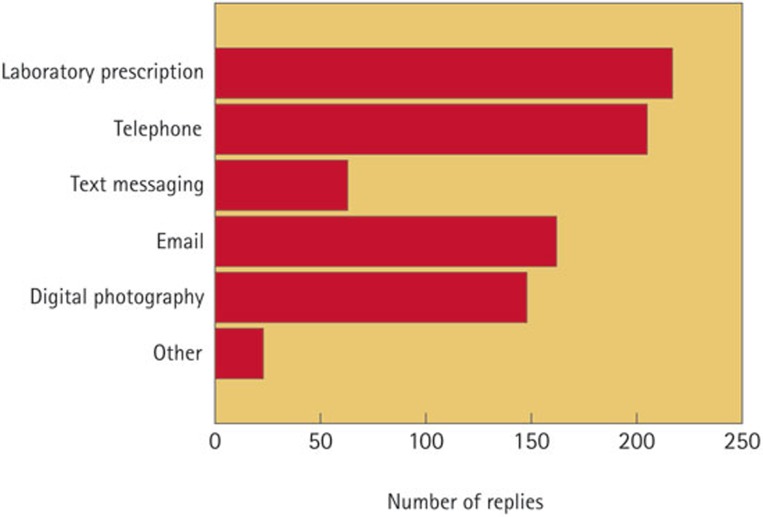
Bar chart showing the methods of contact used by dentists in communicating with the dental laboratory

The results of this study showed that the laboratory prescription and the telephone were the main communication tools used between dentists and dental technicians. Digital means, whether by e-mail or photography, also played an important role (Fig. 1). Statistical analysis of the results showed that a greater number of communication methods were used by large laboratories (Table 2) and that the source of information did not play a significant role.

Almost a quarter of the respondents (24%) indicated that more than half of laboratory prescriptions had an inadequate amount of information on them throughout the course of treatment and 13% had to contact the dentists for further information. The two most frequently missing items from the laboratory prescription were the shade and the date required. These results were not influenced by the size of the laboratory or the source of information, with the exception of the responses about contact with the dentist for further information (p = 0.002). This was more common in the group providing information from records, where 22% reported having to contact the dentist over half the time, compared to only 10% in the memory group. Also, mid-sized laboratories reported a greatest percentage (79%) for shade missing compared (p = 0.01) to the two other groups (60%).

Some of the additional comments in this section of the questionnaire indicated that the need for further communication was time consuming, with the dentist often being difficult to contact during normal surgery hours and also stressed the fact that some prescriptions were illegible or were not fully completed but had additional comments written on them such as 'see e-mail' or 'I will call you to discuss' or 'please call me'.

Regarding shade selection and communication, the results of this study showed that the majority of respondents (73%) received a single shade for over half of the cases and 81% rarely (0-25% of cases) received any photographs with the patient's teeth and shade guide. Only a minority of dental technicians (9%) reported regularly seeing patients for shade matching. Statistical analysis showed that these results were not influenced by the source of information. A statistical significance (p = 0.02) was detected between the size of lab and a single shade chosen. Large laboratories were more likely to receive instruction for a single shade. However, with regards to sending a patient to the laboratory for shade taking there was a negative correlation (p = 0.02) suggesting that larger dental laboratories were less likely to see patients for shade taking.

The last section on communication pertained to time and management issues. Sixty-eight percent of technicians replied that the dentist allowed sufficient time for fabrication of the definitive prosthesis and its return to the dental practice. The majority (74%) of dental technicians felt that they were either completely or partly involved in the dental team. However, one-quarter of the respondents (26%) felt either rarely involved or not at all. A number of respondents seized the opportunity to further comment and the following is a small selection:

'*On the whole communication has got better but I feel the laboratory must make a stand with their clients to get the best treatment for both the patient and themselves*.'

'*I have worked in dentistry now for many years and the issue of lack of communication in the dental team has been a continuing one, which never seems to be resolved*.'

'*I feel that dentists need to realise how valuable the technician's experience and knowledge are, and include them as part of the team and not consider them as a personal servant*.'

*'Would like a bit more appreciation shown!!!!!!*'

'*By far my happiest clients with the happiest patients are the ones that communicate with the laboratory and view it as part of a team effort to achieve the right result for the patient*.'

'*I feel that in general dentists think of us as an afterthought, not really appreciated. Just a thank you now and again would be nice*.'

'*The main problem occurs when it is necessary to speak to the surgeon and he is unavailable due to surgery*.'

'*Technicians should attend more lectures and courses with the dentists to appreciate the dentist's point of view and exchange opinions and ideas*.'

'*I am a laboratory owner and communicate with dental surgeons on a frequent basis. I find my contact to be almost invariably friendly and professional*.'

'*Private clients value technical support and involvement. NHS customers just tell me they want a crown that “drops on” and is completely clear of the occlusion*.'

'*Most surgeons give plenty of time, but some only give 1 week when it arrives in the lab after 2 days in the post*.'

'*I feel that the dentists do not check their impressions*.'

## Discussion

The purpose of this cross-sectional study was to identify the communication methods and production techniques used by dentists and dental technicians for the fabrication of fixed prostheses within the UK from the dental technicians' perspective. The current publication reports on the communication methods and team issues. The last similar UK study was published in 2009.^12^

The response rate was 32%, which falls into the range of other published surveys of dental laboratories.^10^,^11^,^12^,^19^ The difference with the current survey was the fact that it was administered online, in the hope to make it more appealing and easy for respondents.^20^,^21^ Nevertheless, it has been shown^22^ that web and postal surveys yield similar response rates if certain protocols are followed. A limitation of the current survey was the fact that no distinction was made between those laboratories providing a fully private service, a fully NHS service or a mixed arrangement. These diverse cohorts might be experiencing different communication issues and might be utilising alternative fabrication methods. Juszczyk *et al*.^10^ in their 2009 survey used the same DLA database and reported that the majority (61%) of dental laboratories reported doing a mixture of NHS and private work. A previous study^6^ looking at the quality and prescription of single crowns in Wales reported more problems with NHS compared to privately funded work, but no statistics were possible due to the limited sample size.

The majority of the information used to answer the survey questions were sourced by memory. Most of the published surveys have not focused on this issue with the exception of Hatzikyriakos *et al*.^11^ who reported a similar finding, and a previous survey^23^ where it was anecdotally reported that most of the answers were sourced by financial records. The use of memory may introduce personal bias and thus affect the accuracy of the information. The dental technicians could have exaggerated the degree of lack of information and unsatisfactory work received from the dentists. Similarly their personal bias could have affected the responses on their own laboratory procedures. In this study, however, the statistical analysis showed that the method of resourcing information did not play a significant role.

The results of this study showed that the main method of communication between dentists and dental laboratories is still the written prescription and telephone contact. This is in agreement with the previous UK-based survey.^12^ Statistical analysis of the results showed that a greater number of communication methods were used by large laboratories and this is the first time that this has been reported. This could be a reflection on the degree of knowledge on the use of different communication methods within a larger group, or the need to have multiple modes of communication because of the logistics of maintaining contact with a larger number of dentists.

The results of this study showed that in approximately half of the cases the laboratory prescription was lacking important information. This reaffirms the findings of past surveys.^6^,^9^,^10^ The statistical analysis also revealed that the group that based their answers on records reported even higher percentages for the need to obtain further information from the dentist, compared to technicians basing their answers on memory. This implies that the problem might have been under-reported in this survey. The two most common items missing from the written prescription were the 'shade' and 'date required', which is in conflict with the results reported by Stewart^13^ who reported the absence of the 'departmental clinic' and the 'name of the prescribing dentist' as the most frequent omissions. Statistical analysis of the results indicated that there was no statistical association between the size of the lab and the items missing from the prescription except for shade (p = 0.01). The analysis suggested that mid-sized laboratories reported the highest percentage of missing values (79%), with the percentage only around 60% in the two other groups.

Shade selection may be a quite complex and individualised procedure^24^ yet this survey showed that dentists regularly chose a single shade for most of the cases. The statistical analysis also showed that this was most common with large laboratories. No other UK studies reported on this parameter. This is consistent with the findings of Jenkins *et al*.^6^ in Wales, and Hatzikyriakos *et al*.^9^ in Greece who reported that a single shade tab was chosen 50% of the time. A useful adjunct to the written prescription would be a photograph of the tooth in question, ideally with the shade tab placed adjacent to it,^14^,^19^,^24^ but the extent of use has not been previously reported. This simple accessory measure, however, was only occasionally used according to the responses in this survey. If the dentist is not confident in shade matching, and is not prepared to use other measures such as photographs, an alternative solution would be to send the patient to the dental laboratory.^7^,^24^ Alternatively, some dentists arrange for the technician to visit the practice and meet the patient. However, the results showed that this method of communication was not a popular one. Statistical analysis showed that technicians working in large laboratories were less likely to see patients for shade taking (p = 0.02). No other comparable research data was sourced on the frequency of dental technician shade taking in the UK. However, in a study conducted in Greece^11^ almost 30% of shade selection was undertaken by the dental technicians. A possible explanation of the different results might be that the dental laboratory is not conveniently accessible for the patient, which further strengthens to use of photography as an aid to shade matching. Nowadays, many dentists within the UK use a postal service to send the impressions/casts to dental laboratories some distance from the practice.

The vast majority (68%) of the dental laboratories felt that the dentist did allow them adequate time to complete the fabrication of the crown or bridge to the best of their ability and to return it back to the dental practice. This is in contrast with two previous studies in Greece^11^ and Ireland,^8^ which reported that the majority of dental technicians thought that they were pressured for time. Undergraduate training rarely involves the student undertaking any fixed prosthetic laboratory procedures and as a result the dentist may fail to understand the complexities of manufacture and especially the time required.

The UK study by Juszczyk *et al*.^12^ reported that '54% of dental technicians working in a commercial laboratory did feel an integral part of the dental team'. In this survey 22% felt completely involved, the majority of 52% feeling partly involved. The questionnaire allowed the dental laboratory to pass on any additional comments on the survey title. In general the comments indicated that communication methods have improved but there are still many unresolved issues.

A number of papers^1^,^14^,^25^ have recommended that dental school curricula should reinforce the teaching of both the technical stages of laboratory fabrication as well as proper dentist-technician communication in order to ensure high quality team working later on. This has been recognised at Griffith University in Australia^17^ with the introduction of formalised inter-professional education between students of dentistry, dental technology, dental therapists and hygienists. The adoption of similar changes in the curricula of UK dental schools would be recommended. One more way of strengthening communication may be through organising more continuous professional development courses with participation from both parties encouraged.

## Conclusions

Within the limitations of this UK-based study, the following conclusions could be drawn:

1. The main methods of communication between the dentists and dental laboratories are written prescriptions and telephone contact. Newer technologies such as digital photography and e-mail are playing an increasing role
2. The number of communication methods used by laboratories is directly related to their size
3. The laboratory prescriptions often lack important information, such as shade. When shade was prescribed, it was usually a single tab
4. Dental laboratories were, in the main, content with the time allocated for the prescribed work to be fabricated
5. The majority of dental laboratories felt that they were part of the dental team, but there were still some elements of dissatisfaction that need to be improved upon.

## Figures and Tables

**Table 1:** Relevant subchapters of the questionnaire with answers in percentages in parentheses

**GENERAL INFORMATION**1. Please indicate the source of the information that you will be giving:From memory (81%) From records (19%)2. This survey is anonymous so please indicate the country that you are based in:England (90%) Scotland (4%) Northern Ireland (1%) Wales (5%)3. Approximately, what number of dentists do you currently work with?1–25 (43%) 26–50 (30%) 51–75 (8%) 76–100 (6%) 100+ (13%)**COMMUNICATION METHODS**4. Please select all the methods of contact used by dentists to communicate with you:Laboratory prescription (98%) Telephone (93%) Text messaging (29%) Email (73%) Digital photography (67%) Other (10%)Please add any relevant comments5. With regards to the laboratory prescriptions for fixed restorative work, what percentages have an inadequate amount of information on them throughout the course of treatment?0-25% (54%) 26-50% (22%) 51-75% (16%) 76-100% (8%)6. What percentage of laboratory prescriptions do you have to contact the dentist to obtain further information?0-25% (65%) 26-50% (22%) 51-75% (8%) 76-100% (5%)7. Please indicate the two most common items missing from the laboratory prescription when received from the dentist.Patient's name (6%) Shade (75%) Date required (60%) Material to be used (32%) Tooth notation (18%) Other (9%)Please add any relevant comments**SHADE MATCHING**25. What percentage of the time is a single shade (for example, A3 or B2) specified for crown and bridgework?0-25% (7%) 26-50% (20%) 51-75% (33%) 76-100% (40%)26. What percentage of dentists would send you a photograph of the patient's teeth with the shade tab to help you with shading?0-25% (81%) 26-50% (10%) 51-75% (7%) 76-100% (2%)27. What percentage of dentists would send a patient to you to do the shade matching?0-25% (75%) 26-50% (15%) 51-75% (9%) 76-100% (1%)**TIME & TEAM MANAGEMENT ISSUES**28. Do you feel that the dentist generally allows you adequate time to complete the fabrication of the crown/bridge to the best of your ability, and return it to the dental practice?Yes (68%) No (32%)Please comment29. How involved do you feel as part of the dental team?Completely involved (22%) Partly involved (52%) Rarely involved (24%) Not involved (2%)30. Please add any further comments that you may have on the communication between the dentist and laboratory:(63 additional comments)

**Table 2:** Fisher's exact test relating size of laboratory to communication methods used

Lab size	Small (n = 106) Number (%)	Medium (n = 95) Number (%)	Large (n = 47) Number (%)	P-value
Lab prescription	87 (82%)	86 (91%)	44 (94%)	0.08
Telephone	78 (74%)	83 (87%)	44 (94%)	0.004
Text messaging	19 (18%)	26 (27%)	18 (38%)	0.02
Email	50 (47%)	72 (76%)	40 (85%)	<0.001
Digital photography	48 (45%)	60 (63%)	40 (85%)	<0.001

## Notes

**Refereed Paper**
